# The Aachen Treatment of Syphilis

**Published:** 1896-06

**Authors:** 


THE AACHEN TREATMENT OF SYPHILIS.—Dr. J. Bion Bogart read a paper before the Brooklyn Surgical Society (*Brooklyn Medical Journal*) in which he described the Aachen treatment of syphilis (*Medicine*). He says that the temperature of the waters used for therapeutic purposes is from 38° to 72° C., and they contain from 22 to 28 grammes of chloride of sodium, 4 to 5 grammes of sulphites, and 8 to 12 grammes of carbonates, to 10,000 cc. The waters are used for immersion, douche and vapor baths, and are taken internally.

A bath in Aachen of 95° F., of half an hour's duration, makes the skin soft and moist, and the chlorides and bicarbonates contained in the water free it in the simplest and most agreeable manner from adherent epidermic scales; moreover, by the opening of the sebaceous and sweat ducts all obstructing masses of secretion are easily removed. Whilst these circumstances favor the increased excretion of gaseous and fluid substance, both during and after the bath, the skin is also prepared for taking up medicinal substances, which are employed with effect in the course of certain methods of treatment.

The Aachen treatment is the inunction treatment of Sigmund, facilitated and protected by the use of the Aachen waters internally and externally, as immersion, douche and vapor baths. Moreover, during the treatment, the most scrupulous attention is paid to local and general hygiene, and the nutrition of the body is energetically maintained.

Dr. Bernard Brandis (*Geheimer Sanitatsrat*), Aachen, in a paper translated by Hugh A. Auchinleck, of Dublin, in 1881, announced the following "Principles of the (inunction) Treatment of Syphilis:" "The body must always be adequately prepared for the absorption of the mercury; and the gray ointment must always be administered carefully and in sufficient quantity. The body must, during the treatment, be preserved sound. The inunction treatment must be carried out long enough."

The principles of the Aachen treatment cannot be better stated.

The preparations used for the inunction is the Unguentum Hydrargyri Cinereum of the German Pharmacopœia, which differs from ours by containing one-third less mercury and twice as much lard as suet, while ours contains these two ingredients in equal amounts. The German preparation is, therefore, weaker and softer, both of which qualities make it more suitable for inunction. Another very important point is that the ointment is always freshly prepared; hence less irritating to the skin and better borne by the system in general.

To meet the first indication, that "The body must always be adequately prepared for the absorption of the mercury," an immersion bath of 95° F. of half an hour's duration is usually all that is necessary, if we remember the remarkable effects of the Aachen waters in softening and cleansing the skin. The bather sits on a marble seat with the body completely submerged. Soap is not required, and, as a rule, none is used except after each course of inunctions to prepare for the next. Immediately after the bath the patient is dried, and the inunction follows in the order laid down by Sigmund: "On the first day rub both legs; on the second, both thighs; on the third, abdomen and breast; on the fourth, the back; on the fifth, both arms." In Aachen the sides of the body take the place of the abdomen and breast in Sigmund's formula.

The amount of ointment used at each inunction varies in the adult from 4 to 10 grammes (1 to 2½ drachms). The rubbing lasts twenty minutes, and is done by experienced rubbers, who use both hands, unprotected, simultaneously. These men often give from ten to fifteen treatments for several days in succession, yet seldom experience any ill-effect from the drug.

Three or four glasses of the water are drank daily during the entire course of treatment, generally before breakfast.

The vapor bath is generally ordered when the patient not only ceases to improve, but also exhibits a return of symptoms previously subdued, or new phases of the disease make their appearance. These phenomena are interpreted as indicating that the mercury is no longer active. In such cases vapor baths are usually given on three successive days, the inunctions being meanwhile interrupted. Afterward, a vapor bath is generally administered every tenth day to guard against a recurrence of these symptoms.

The use of atropine in syphilitic iritis; the local application of mercurial plasters over painful areas, glandular and bony swellings, and for various syphilides of the skin; antiseptic lotions, douches and dressings; and the sharp spoon and scalpel—all find their appropriate places as adjuncts to the "Aachen treatment." The use of cutting instruments is, however, limited to suppurating and ulcerative processes.

One of the most striking features of the Aachen cure is the comparatively insignificant role which it assigns to the iodide of potassium. This drug is looked upon as for the most part a symptomatic remedy; for, while its marvellovs power to relieve pain and ameliorate certain symptoms of a distressing and often dangerous character is freely acknowledged and frequently taken advantage of, it appears to be almost universally distrusted as a curative agent. Perhaps this fact cannot be better illustrated than by the following quotation from an article upon the Aachen treatment by Drs. Brandis and Schumacker: "But whilst recognizing the magical results produced by iodide of potash, we must not forget that experience teaches that the worst lesions only slumber during its administration, and we must not be betrayed by its power of causing the disappearance of symptoms into the belief that the disease has been extinguished. The early stages of central nervous disease, especially commencing tabes, with its paralyses, which may quickly disappear on the exhibition of iodide of potash, often prove the deceitful nature of the remedy by a later and severe outbreak of the disease."

The treatment may be employed in all cases where relief is possible, and no individual peculiarity and no time of life makes an exception.

Experience teaches that the earlier symptoms may extend over a period of years, but suitable treatment and careful watching will bring the majority of patients to the wished-for goal of perfect recovery within from one to three years, while the recurrence of the earlier signs of the disease seven or eleven years after infection, which we have sometimes seen, is to be regarded as exceptional.

An exemption of at least two or three years from the earlier manifestations of syphilis must precede marriage.—O. D., in *St. Louis Med. & Surg. Journal.*

---

A NEW TEST FOR SUGAR.—A new test for sugar in the urine is made by dissolving 2 gme. of salicylate of sodium, 2 gme. of salicylate of copper, and 8 grm. of sodium-carbonate crystals in 100 c. c. of distilled water. Add an equal volume of the reagent to the urine to be tested, and apply heat until a precipitate is thrown down. The presence of glucose will be indicated by a yellow deposit, the suboxide; and the contrary will be shown if the precipitate is of a grayish or black color, representing the binoxide.—*American Medical-Surgical Bulletin.*

